# Droplet nuclei caustic formations in exhaled vortex rings

**DOI:** 10.1038/s41598-022-07717-z

**Published:** 2022-03-10

**Authors:** Andreas Papoutsakis, Ionut Danaila, Francky Luddens, Manolis Gavaises

**Affiliations:** 1 Department of Mechanical Engineering and Aeronautics, School of Mathematics, Computer Science and Engineering, City University of London, London, EC1V 0HB UK; 2 Laboratoire de Mathématiques Raphaël Salem, Université de Rouen Normandie, CNRS UMR6085, 76801 Saint-Étienne-du-Rouvray, France

**Keywords:** Biomedical engineering, Physics, Fluid dynamics

## Abstract

Vortex ring (VR) structures occur in light or hoarse cough configurations. These instances consist of short impulses of exhaled air resulting to a self-contained structure that can travel large distances. The present study is the first implementation of the second order Fully Lagrangian Approach (FLA) for three-dimensional realistic flow-fields obtained by means of Computational Fluid Dynamics (CFD) and provides a method to calculate the occurrence and the intensity of caustic formations. The carrier phase flow field is resolved by means of second order accurate Direct Numerical Simulation (DNS) based on a Finite Difference approach for the momentum equations, while a spectral approach is followed for the Poisson equation using Fast Fourier Transform (FFT). The effect of the undulations of the carrier phase velocity due to large scale vortical structures and turbulence is investigated. The evaluation of the higher order derivatives needed by the second order FLA is achieved by pre-fabricated least squares second order interpolations in three dimensions. This method allows for the simulation of the clustering of droplets and droplet nuclei exhaled in ambient air in conditions akin to light cough. Given the ambiguous conditions of vortex-ring formation during cough instances, three different exhale (injection) parameters *n* are assumed, i.e. under-developed ($n=2$), ideal ($n=3.7$) and over-developed ($n=6$) vortex rings. The formation of clusters results in the spatial variance of the airborne viral load. This un-mixing of exhumed aerosols is related to the formation of localised high viral load distributions that can be linked to super-spreading events.

## Introduction

Respiratory infections can be transmitted through droplets ($>5\;\upmu$m) and droplet nuclei ($<5\;\upmu$m)^1–3^. According to the World Health Organization (WHO) report^2^ COVID-19 in particular, is primarily transmitted through droplets and contact^4–6^. The airborne route is a potentially important transmission pathway for viral infection in indoor environments since contact transmission can be limited by fast inactivation of the virus on hands. Furthermore, contact transmission often incorporates an initial airborne path where large inocula droplets deposit on surfaces through sneezing^7^. The interaction of the carrier phase flow field with the exhaled droplets dictates the intensity of the viral load and the infection distance. Recent research^8–10^ has shown that Vortex Rings (VRs) can be produced in coughs and can enhance the transport of fine cough droplets. Vortex ring structures occur in light or hoarse cough configurations^8^. These instances consist of short impulses of exhaled air result to a self-contained structure (i.e., vortex ring) that can travel large distances. Increased concentrations of airborne viral loads have been related to super-spreading events^11^, and have been attributed to individual behaviour of coughing and sneezing^12^ and also to specific activities^13^.

Turbulence increases the mixing of droplets and droplet nuclei^14^ and the settling velocity^15,16^. As the droplet size and Stokes number increase, the particle trajectory memory becomes pronounced; thus, un-mixing^17,18^ and Random Uncorrelated Motion (RUM)^19^ are observed. Un-mixing results in the segregation of droplets by a preferential concentration, clustering mechanism^20^.

Clustering is exhibited as the formation of narrow local droplet accumulation regions in the zones of low vorticity and high strain rate^21^ and have been associated with zero-acceleration points^22^. For high Stokes number regimes, inertia droplets sample the carrier phase velocity field as a white noise^23^, forming clusters due to a multiplicative amplification mechanism^23–25^. This multiplicative process of amplification and dilatation was identified^19^ as the deformation of the Lagrangian volume of the dispersed phase^26^ transported along a particle trajectory. The Lagrangian volume may vanish, giving rise to instantaneous singularities in the particle concentration field identified as caustics or caustic formations. In addition to mixing and un-mixing (clustering), RUM was identified as a third type of characteristic response of the dispersed phase in turbulent flows, applicable to all Stokes numbers^19^. The occurrence of RUM was linked with the occurrence of singularities due to trajectory intersections related to the trajectory history. Particle motion, as the overlapping of a mesoscopic smoothly varying component and RUM, was identified in^27–29^. RUM results in a multi–valued velocity field of the dispersed phase due to the folding of the dispersed continuum, playing a significant role in collision processes.

The intensity of caustics is important to a great variety of environmental, biological, engineering and cosmological^30–34^ flows (e.g., environmental pollution^35^, the impact of radioactive particles^36^ and the potency of spray delivered medication^37^ are dependent on the formation of such accumulation regions^38^), and has become the focus of experimental^39^ and theoretical work^40^. The Fully Lagrangian Approach (FLA or Osiptsov method)^41^, treats the particulate phase as a pressureless continuum^40^, by introducing the Jacobian matrix determinant $|{{\mathbf {J}}}|$ (i.e., $\rho _0=|\mathbf {J}|\rho$)^42^. Thus, continuous fields for the particle number density and the particle velocity can be defined on the Lagrangian dispersed continuum. Due to the absence of pressure, the continuum can intersect itself creating overlapping folds. Particle collisions may occur among particles that are attached to overlapping folds^43^, thus a low particle loading constrain needs to be incorporated in order to support the pressureless continuum assumption. The use of the FLA in turbulent flows^19,44^ resulted in the identification of the concentration distribution and the identification of the mechanisms involved in the segregation process^18,19^. A turbulent diffusion model was introduced for the first order FLA, implemented for numerical experiments in homogeneous isotropic turbulence and assessed in comparison with DNS simulations using a standard Lagrangian approach for the dispersed phases^45^. In the same paper it was also reported that the singularities of the point-wise number density inferred from the standard FLA pollutes spatially averaged results. A second order closure for the spatially filtered number density was then presented, in which the FLA number density was interpreted alongside with the spatial structure of the dispersed continuum, and was connected to a length scale^40^.

The introduction of the FLA into the study of turbulent flows^44^ resulted in the identification and analysis of spatial structures of the dispersed phase distribution using the moments of concentration. FLA analysis concluded that particle concentration in the long term follows a log normal law^19^. This is consistent with an alternative analysis using Voronoï tessellation^46^. Also, FLA studies on DNS of homogeneous and isotropic turbulence led to identification of the mechanisms involved in the segregation process^18,19^. Using the FLA in turbulent flows also enabled the quantification of the singularities related to trajectory intersections and the establishment of a relation between the frequency of their occurrence and the Stokes number.

In this paper we present the first implementation of the second order Fully Lagrangian Approach (FLA2) for three-dimensional realistic flow-fields obtained by means of Computational Fluid Dynamics (CFD). We provide a method to calculate the occurrence and the intensity of caustic formations. This is achieved by calculating a principal direction of the caustic front based on the tensor of the Hessian of the deformed continuum. The structure of the paper is as follows. The range of the conditions and the configuration of the cases studied are presented in the “Results” section. In the same section the FLA and the FLA2 results are presented and discussed. Specifically, the solution of the number density for the exhaled Lagrangian clouds is presented for low Reynolds axisymmetric and also high-Reynolds fully turbulent configurations, for three distinct exhale parameters. In the “Methods” section the implementation of the FLA2 for CFD configurations is presented and the results are further discussed in the “Discussion and conclusions” section. The DNS methodology and the implementation of the second order FLA for three-dimensional turbulent flow fields is presented in the “Supplementary Material”.

## Results

In order to investigate the occurrence of caustics of droplet nuclei in coughs due to large scale vortical structures and due to turbulence we performed a series of DNS of cough configurations. The scope of this analysis is the calculation of the droplet distribution, the investigation of the occurrence of caustics and finally the calculation of the intensity of the droplet nuclei clustering. The exhaled droplet nuclei distribution is modelled by representative droplet clouds, integrated in time using a standard Lagrangian approach based on the fully resolved carrier phase flow field. The number density of the droplet nuclei for each cloud is calculated using the Fully Lagrangian Approach^26,47^ Although the standard FLA identifies the occurrence of caustics, the second order FLA is used to calculate the intensity of the caustics by providing a measure of the number density filtered at a given length-scale $R_\varepsilon$ as ${\hat{n}}_d^{R_\varepsilon }$. The numerical implementation of the method is described in detail in the “Supplementary Material” of this paper.

### Cases setup

Given the ambiguous conditions of the human coughs as described in literature^8,10,48,49^ we focus in simulations to carrier flows represented by vortex rings with Reynolds and Stokes numbers related to human cough conditions. Vortex rings are produced by the sudden injection of exhaled air in a surrounding flow that is initially at rest. Theoretical description of vortex rings^50^ generally assumes that the flow injection producing vortex rings is generated by a piston-cylinder mechanism. Using this equivalence, we consider injection pulses with a finite duration *T* and average injection speed $U_0$, issuing from an orifice of diameter *D*. The exhale (or injection) parameter is then $n=T U_0/D$, corresponding to the piston stroke ratio in a piston-cylinder mechanism. Long duration of exhumation impulses, as the ones observed in a typical cough, result to large injection parameters ($n>40$), which lead to the formation of jets. The exhale parameter *n* is related to the temporal injection profile *U*(*t*) (or injection program) and the average injection velocity $U_0$ as:

1 $$\begin{aligned} n=\frac{1}{D} \int _{t=0}^{t=T+\tau } U(t) dt = \frac{U_0 T}{D}, \; \text {with} \; \frac{U(t)}{U_0}= {\left\{ \begin{array}{ll} 3\left( \frac{t}{\tau }\right) ^2-2\left( \frac{t}{\tau }\right) ^3, t< \tau \\ 1 , \tau< t< T \\ 3\left( \frac{T -t}{\tau }\right) ^2-2\left( \frac{T -t}{\tau }\right) ^3, T< t< T + \tau \\ 0, T + \tau < t \end{array}\right. }. \end{aligned}$$

Injection velocity program (1) has a trapezoidal shape, with acceleration and deceleration ramps of duration $\tau$. We set in all simulations $\tau =0.1 T$.

**Figure 1:**
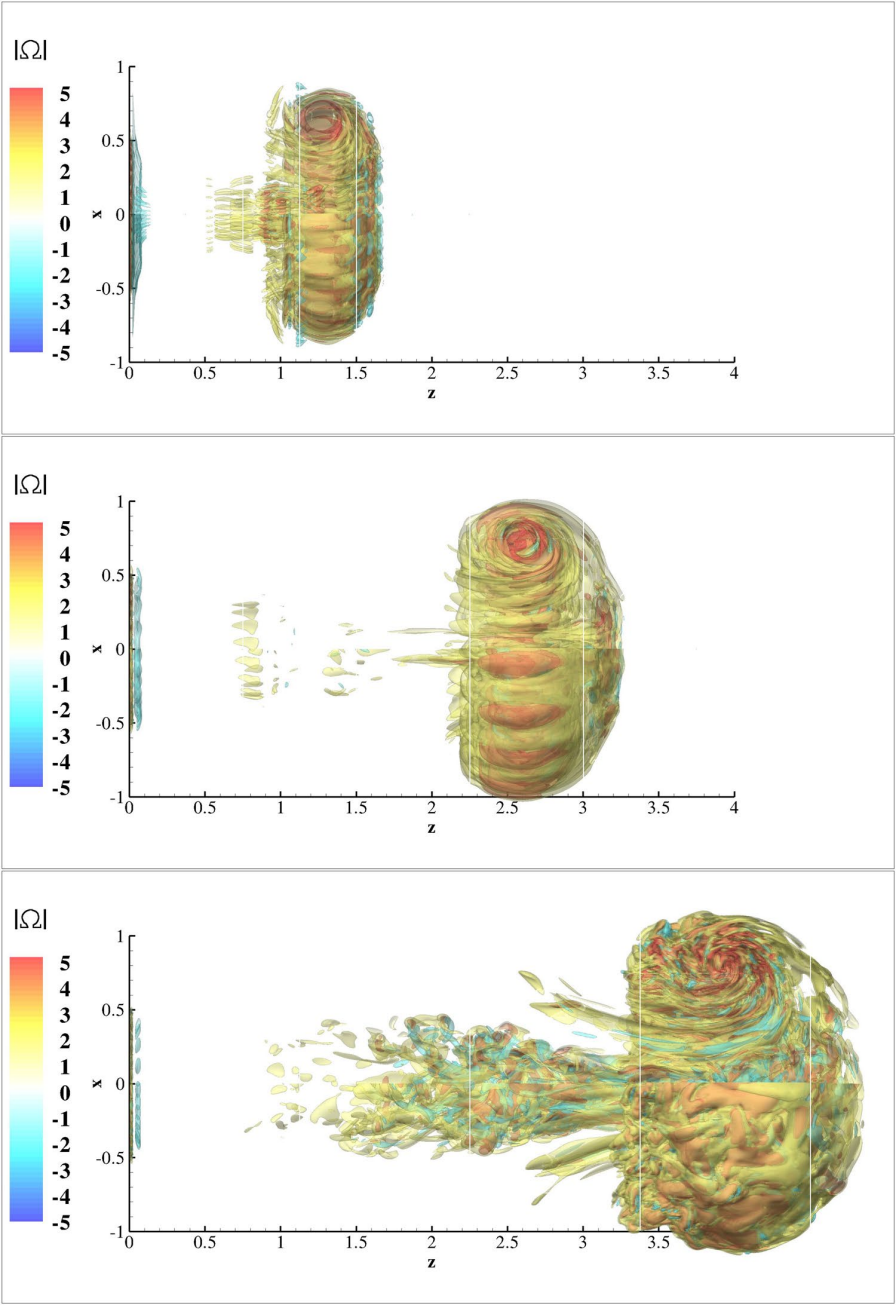
Non-dimensional vorticity magnitude $|\Omega |$ iso-surfaces, normalised with $U_0^2/L$ for the test cases C3DN2 at $t=4 \times 10^3$ s (top) C3DN4 $t=8 \times 10^3$ s (middle) and C3DN6 (bottom) at time $t=12 \times 10^3$ s equal to twice the injection period *T* for each one of the cases simulated.

A coarse estimate for the exhale parameter *n* of a cough can be derived from the volume *V* of the air exhaled. Following the classical slug-flow approximation, we assume that $V= (\pi D^2/4) T U_0$ and obtain $n=4V/\pi D^3$. The typical exhaled volume from a cough has been calculated in the range of 0.25–3 l^9,51^. Thus, the exhale parameter for coughs at this exhaled volume range spans from $n=40$–477, assuming a mouth opening of $D=2$ cm. For such high exhale/injection parameters, the injected flow consists of a leading vortex ring followed by a trailing jet. It was observed^52^ that the vortex ring carries only a fraction of the circulation produced by the injection process, the rest being injected in the trailing jet. The critical time at which the vortex generator produces the amount of circulation engulfed by the vortex ring was called *formation time* and the corresponding injection parameter (or stroke ratio) formation number F. The value of *F* depends on different injection parameters and ranges from 1 to 8. For laminar injections, with trapezoidal injection profiles, as in the present study, it was experimentally found^52^ that $F \approx 3.6$–4.5. This means that for our injection program (1), if $n <4$ the full injected slug will be absorbed by the vortex ring, while for $n>4$ the vortex ring will be followed by a visible trailing jet. Higher is *n*, longer is the trailing jet. Given the evidence of vortex rings in intermittent coughs^8–10^, we focus in the narrow region of exhalation regimes characterised by low injection/exhale parameters *n*. This choice is also supported by the observation that the entrainment of the surrounding air in the flow is mostly due to the high circulation of the vortex ring, which is thus expected to mainly contribute to the long-distance propagation of particles. We consider, however, three different injection/exhale parameters *n*: two values, $n=2$ and $n=3.7$, lower or equal to the formation number *F*, corresponding to vortex rings without trailing jets, and the value $n=6$ for a vortex ring with a substantial trailing jet (see Fig. 1).

The nominal exhale velocities range between $U_0=1$ m/s to $U_0=10$ m/s which include experimentally measured coughs^8,53^ and breathing scenarios^3^. The value of the injection Reynolds number $Re=U_0 D/\nu$ was reported^3,51^ to range between $Re=1000$ and $Re=16$,000. A synopsis of the aerodynamic conditions of coughs as presented in the literature is shown in Table 1. A typical value^51^ of the orifice diameter is 2 cm, while a reasonable choice of the air kinematic viscosity is $\nu =15.6 \times 10^{-6}\,{\text {m}}^2/{\text {s}}$. In our simulations we selected two representative set of cases characterised by a low Reynolds number $Re=1000$ (axisymmetric laminar simulations) and a high Reynolds number set of cases with $Re=10$,000 (three dimensional simulations).

**Table 1:** Flow conditions for cough in literature.

Author	*u* (m/s)	*D* (cm)	*V* (l)	Droplet nuclei *d* ($\upmu$m)
Verma et al.^10^	2–6	–	$> 0.5$	5–10
Bourouiba et al.^51^	–	2	0.25–1.6	10
Tang et al.^53^	2–25	2	1–3	–
Liu et al.^9^	20	2.26	1	5–100

For the droplet size distribution used in our simulations we used eight bins of representative particles with diameters from 0.5 to $100\;{\upmu }$m in agreement to droplet nuclei sizes reported in literature^3,49,51^. The laminar axisymmetric simulations $Re=1000$ correspond to $U_0 \sim 1\;m/s$ and a characteristic time of the flow $t_0=D/U_0 \sim 20 \times 10^{-3}$ s, the fully turbulent three-dimensional simulations $Re=10$,000 to $U_0 \sim 10$ m/s and $t_0 \sim 2 \times 10^{-3}$ s. For the three-dimensional turbulent simulations the sinusoidal fluctuations described in the “Supplementary Material” were superimposed on the inlet velocity program in 1, assuming $k_T=0.1$ and $\varepsilon _T=1$. The details of the transitional and fully turbulent simulations are presented in Table 2.

**Table 2:** Cases simulated.

Name	Domain Size	$n\theta \times nr \times nz$	T (ms)	n	Re
C2DN2	$2D \times 4D$	$1 \times 512 \times 4096$	4	2	1000
C2DN4	$2D \times 8D$	$1 \times 512 \times 4096$	8	3.7	1000
C2DN6	$2D \times 12D$	$1 \times 512 \times 4096$	12	6	1000
C3DN2	$1.5D \times 3D$	$513 \times 256 \times 512$	4	2	10,000
C3DN4	$1.5D \times 6D$	$513 \times 256 \times 1024$	8	3.7	10,000
C3DN6	$1.5D \times 9D$	$513 \times 256 \times 1536$	12	6	10,000

Cases, C2DN2, C2DN4 and C2DN6 (See Table 2) consist of axisymmetric simulations of exhaled vortex rings that account for the resolution of long injection times and longer transport distances. Each one of these cases corresponds to different exhale parameters resulting to different ratios of droplet nuclei encapsulated within the vortex rings in relation to the particles left behind in the trailing jet. Cases C3DN2, C3DN4 and C3DN6 are three dimensional turbulent simulations of exhaled VRs. The domain length spans for twice the final distance covered by the VR (3D, 6D and 9D) and incorporates non-reflective outlet boundary conditions^54^, thus ensuring a minimal influence to the flow structures. In the radial direction the total domain radius corresponds to a confinement coefficient of 3 which has limited effect on the formation and the circulation of the VR^54^. Grid size resolves the finest turbulent scales ($\Delta x = 0.0039D\sim 2 \eta = Re^{-3/4}D$), where $\eta$ is the Kolmogorov length scale. The low-Reynolds number simulations of vortex rings (Re = 1000) have been carried out by means fully resolved 2d-axisymmetric simulations^54,55^. Although coherent vortical structures emerge they do not cascade to fine three-dimensional turbulent fluctuations. The high Reynolds cases^56^ (Re = 10000) result in turbulent vortex rings integrated by means of Direct Numerical Simulations^45,57^. The cases simulated are of increasing computational cost due to the larger computational domain and long simulation time required when the exhale parameter *n* increases.

The exhaled droplet and droplet nuclei relaxation time $\tau _0$ is defined as:

2 $$\begin{aligned} \tau _0=\frac{\rho _{d} d^2}{18 \mu _{air}}, \end{aligned}$$

where $\rho _d$ is the droplet density, $\mu _{air}$ the carrier phase dynamic viscosity and *d* the droplet size. Droplet sizes simulated in our study are provided^49^ in Table 3. The Stokes number of the particles $St=\tau _0 U/D$, is:

3 $$\begin{aligned} St=\frac{\rho _d}{18 \rho _{air}} D_d^2 Re, \end{aligned}$$

where $D_d=d/D$ is the non-dimensional droplet diameter. Droplet clustering occurs for local Stokes numbers greater than unity $St>1$. Based on the macroscopic timescale $t_0=D/U_0$ a critical diameter $\delta _0$ is defined as:

4 $$\begin{aligned} \delta _0^2 =\frac{18 \rho _{air}}{\rho _{d}} Re^{-1}. \end{aligned}$$

**Table 3:** Droplet sizes and Stokes numbers used in the simulations.

Bin	1	2	3	4	5	6	7	8	$\delta$
d ($\mu$m)	0.5	1	2	5	10	20	50	100	
*St* (Re = $10^3$)	$2.89{\text {e}}-05$	$1.16{\text {e}}-04$	$4.62{\text {e}}-04$	$2.89{\text {e}}-03$	$1.16{\text {e}}-02$	$4.62{\text {e}}-02$	0.289	1.16	$\delta _{0}= 93.0\;{\upmu }$m
$St_t$ (Re = $10^3$)	$9.13{\text {e}}-04$	$3.65{\text {e}}-03$	$1.46{\text {e}}-02$	$9.13{\text {e}}-02$	0.37	1.46	9.13	36.5	$\delta _{\tau } = 16.5\;{\upmu }$m
*St* (Re = $10^4$)	$2.89{\text {e}}-04$	$1.16{\text {e}}-03$	$4.62{\text {e}}-03$	$2.89{\text {e}}-02$	0.116	0.462	2.89	11.6	$\delta _{0} =29.4\;{\upmu }$m
$St_t$ (Re = $10^4$)	$2.89{\text {e}}-02$	0.116	0.462	2.89	11.6	46.2	289.0	1155.0	$\delta _{\tau }=2.94\;{\upmu }$m

The Stokes number is then presented as $St =(D_d/\delta _0)^2$. For turbulent flows, the time scale is $D/U Re^{-1/2}$ and the Stokes number is defined as $St_t=\tau _0 U_0 /(D Re^{-1/2})$ resulting in a critical diameter $d_t$ defined as:

5 $$\begin{aligned} \delta _t^2 =\frac{18 \rho _{air}}{\rho _{d}} Re^{-3/2}. \end{aligned}$$

Droplets with diameter $d>\delta$ are expected to exhibit trajectory crossing^39^. In our simulations we assume a range of droplet nuclei dimensions from 0.5 up to $100\;\upmu$m. Critical droplet diameters $\delta _0$ and $\delta _t$ for the macroscopic and the turbulent time scales are shown in last column of Table 3. In the same table we present the droplet nuclei characteristics for eight distinct representative sets of parcels (bins) issued during the cough simulation. As it can be inferred from Table 3, the selected diameters for the representative droplet nuclei parcels result in a wide range of Stokes numbers from nearly non-inertia droplets (i.e., $\mathrm{{St}}<10^{-3}$) to droplets well within the ballistic regime (i.e., $\mathrm{{St}}>10$)^58^. The particulate Reynolds number ${\mathrm{{Re}}}_d=U_0 d/\nu$ can be expressed as a function of the macroscopic Reynolds number as ${\mathrm{{Re}}}_d=\mathrm{{Re}} \times d/L$, for the regimes simulated (Re = 1000–10,000 and $d=0.5$–$100\;{\upmu }$m it spans from as low as 0.025–50. Although FLA can be applied for non-Stokesian drag law expressions^45^, the specific computational setups for both droplets and droplet nuclei are within the applicability regime of the Stokes law, which is used in this study.

Every $s=10$ computational timesteps, N droplet clouds for each size bin are injected at random positions on the orifice cross-section. The number N of injected clouds per *s* timesteps is:

6 $$\begin{aligned} N=(c A u_r s \Delta \mathrm{t})/n_c, \end{aligned}$$

where *c* is the number density of the exhumed plume, $A=\pi D^2/4$ is the orifice cross-sectional area, and $n_c$ is the number of droplets represented by each cloud which is constant. The particulate velocity is considered equal to the inlet velocity of the carrier phase, and the initial conditions for the Jacobian and Hessian entries are defined as dictated by the FLA (see “Supplementary Material”). Although the number density distribution of droplets and droplet nuclei is well documented in literature^49,51^ in the order of 1–$10 \times 10^6/{\text {m}}^{3}$, in our analysis we focus in the relative compression of the dispersed phase.

### Droplet nuclei distribution

**Figure 2:**
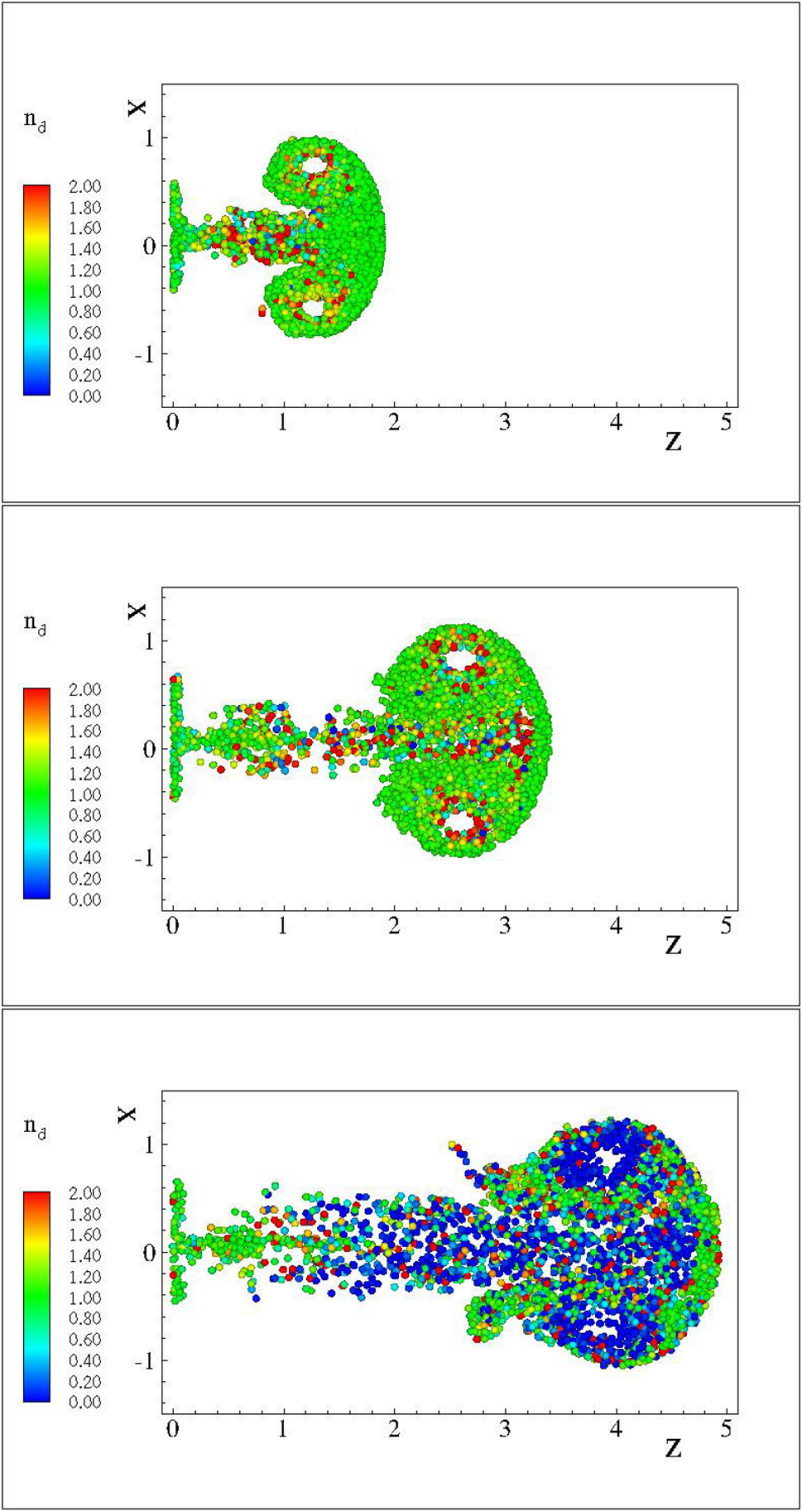
FLA number density for $d=2.0\;\upmu$m droplet nuclei, for the test cases C3DN2 at $t=4 \times 10^3$ s (top) C3DN4 $t=8 \times 10^3$ s (middle) and C3DN6 (bottom) at time $t=12 \times 10^3$ s equal to twice the injection period *T* for each one of the cases simulated.

Iso-surface of vorticity for the three-dimensional cases C3DN2, C3DN4 and C3DN6 are shown in the Fig. 1 at times 2*T* after the end of the injection. For the cases with $n<=3.7$ the flow field vorticity has been almost completely absorbed by the VR. The corresponding distributions of droplets with $d=2\;{\upmu }$m at the same instances are shown in Fig. 2. As it can be inferred form Fig. 2 the vortex rings with $n=2$ and $n=3.7$ present a thin trail of droplets along the trail of the vortex rings. The colour of the scatter points corresponds to the number density inferred from the FLA. For $n=6$ the trailing droplet nuclei are significant and lag the evolution of the VR. This results to the dilation of the trailing droplets as they lag behind, as it can be inferred from the low number densities shown in blue. For all three cases high number densities occur at the re-circulation regions and at the front of the vortex ring.

**Figure 3:**
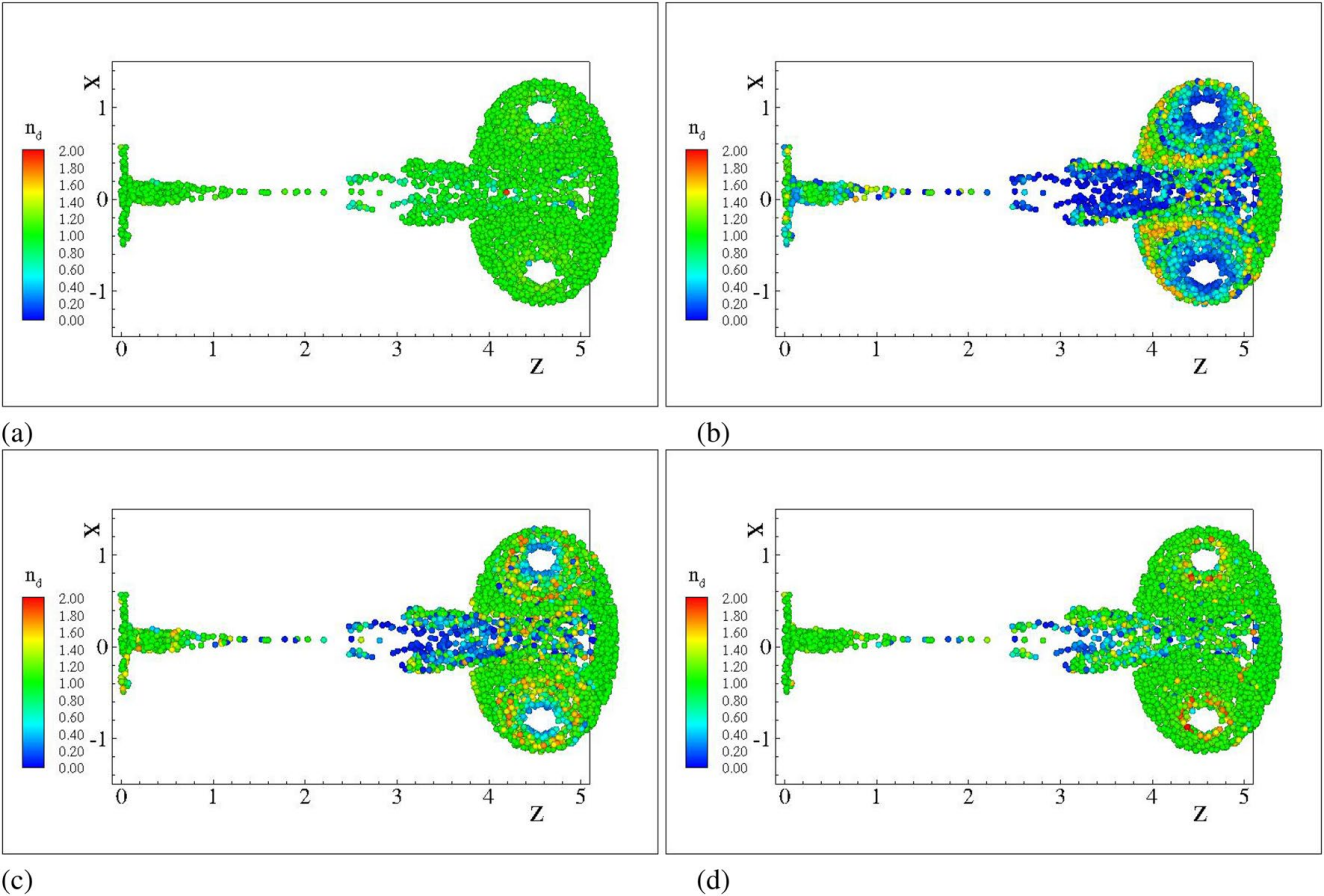
Number density for $d=2.0\;{\upmu }$m droplet nuclei. Case C2DN2. $T=4$ ms (**a**) FLA (**b**) FLA2 $R_\varepsilon =0.01$ (**c**) FLA2 $R_\varepsilon =0.001$ (**d**) FLA2 $R_\varepsilon =0.0001$.

**Figure 4:**
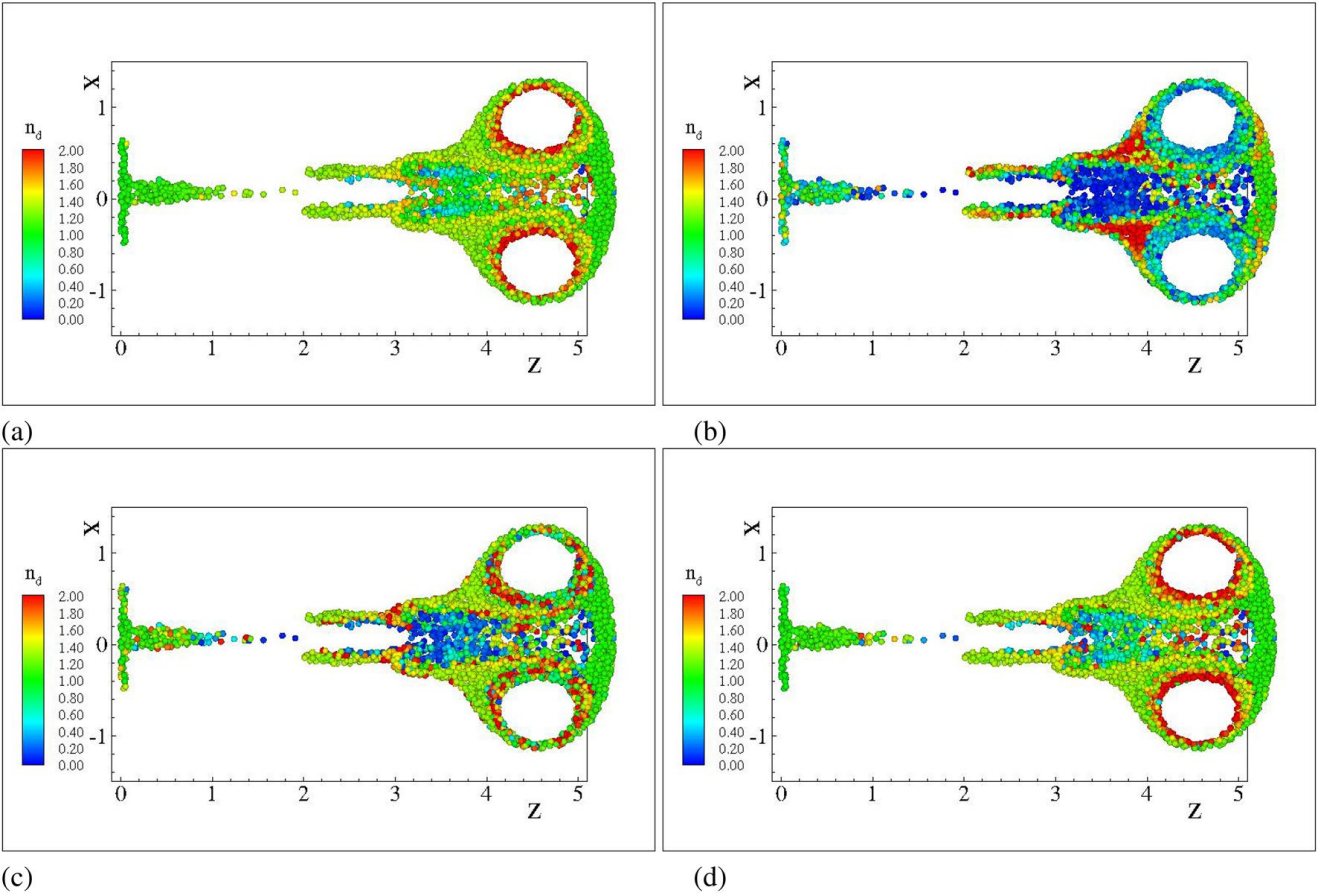
Number density for $d=5.0\;{\upmu }$m droplet nuclei. Case C2DN2. $T=4$ ms (**a**) FLA (**b**) FLA2 $R_\varepsilon =0.01$ (**c**) FLA2 $R_\varepsilon =0.001$ (**d**) FLA2 $R_\varepsilon =0.0001$.

In Figs. 3 and 4 we present the FLA results for the number density and the FLA2 result for various filtering widths $R_\varepsilon$; for droplets with diameters $2\;{\upmu}m$ and $5\;{\upmu}m$, respectively. The filtering widths correspond to different orders of magnitude from $R_\varepsilon = 10 ^{-4}D$ to $R_\varepsilon = 10 ^{-2}D$ which correspond to filter sizes from $2\;{\upmu }$m to 0.2 mm. It can be observed that qualitatively, the solution converges to the FLA solution as $R_\varepsilon$ tends to zero. For the larger filtering lengths the areas in the vicinity of caustic regions are affected, resulting in variations between FLA and FLA2 inside larger areas as the filtering width increases.

### Caustics occurrence

**Figure 5:**
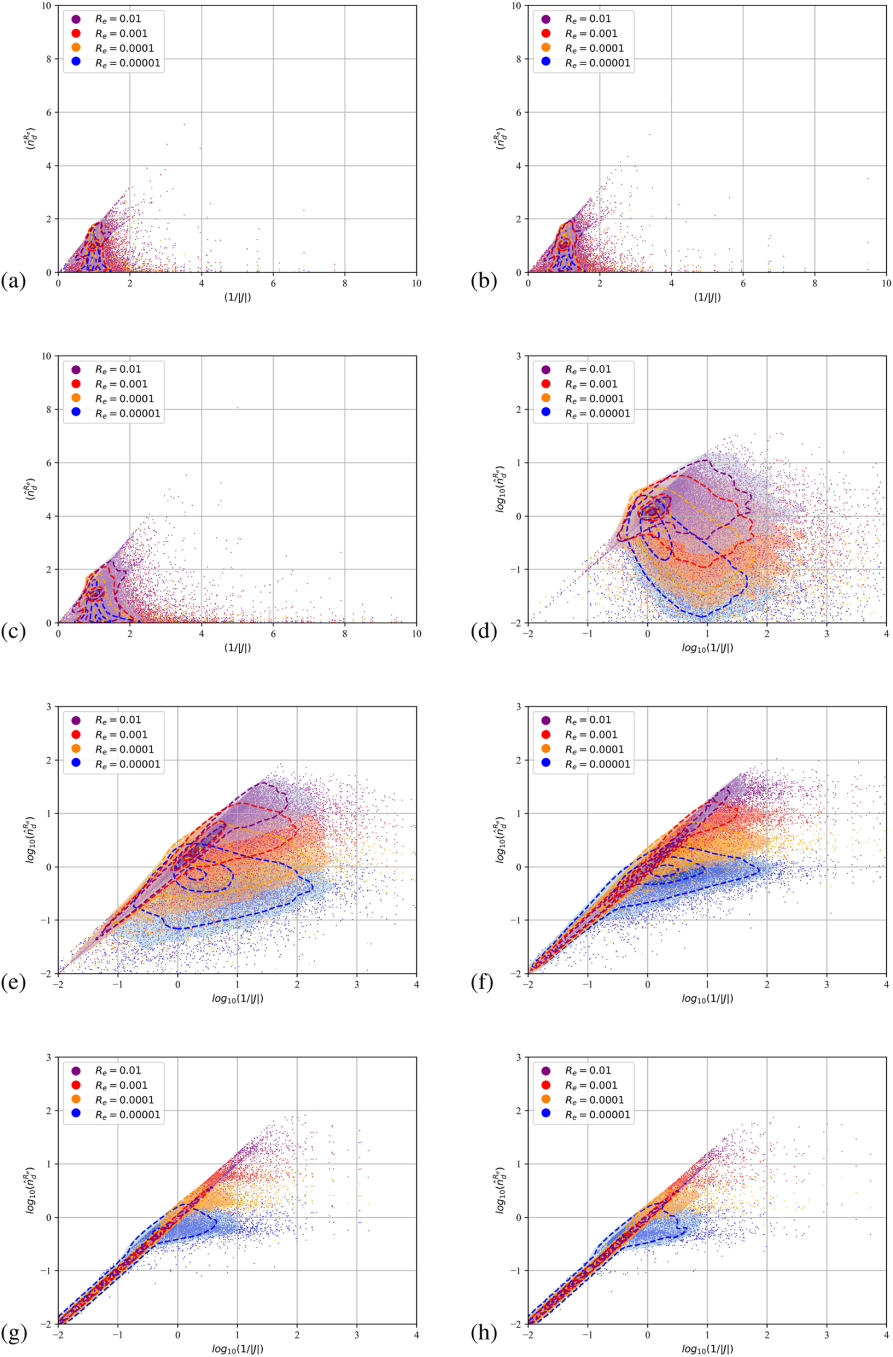
Scatter plot of the filtered FLA2 number density versus 1/*J*. Colours correspond to the filtering length. Dashed iso-lines correspond to the kernel density estimation at levels 10% 50% and 90%. Case C3DN2. $T=4$ ms.(**a**–**h**) $D_d=0.5$–$100\;\upmu$m. (**d**–**h**) are presented in logarithmic scale.

**Figure 6:**
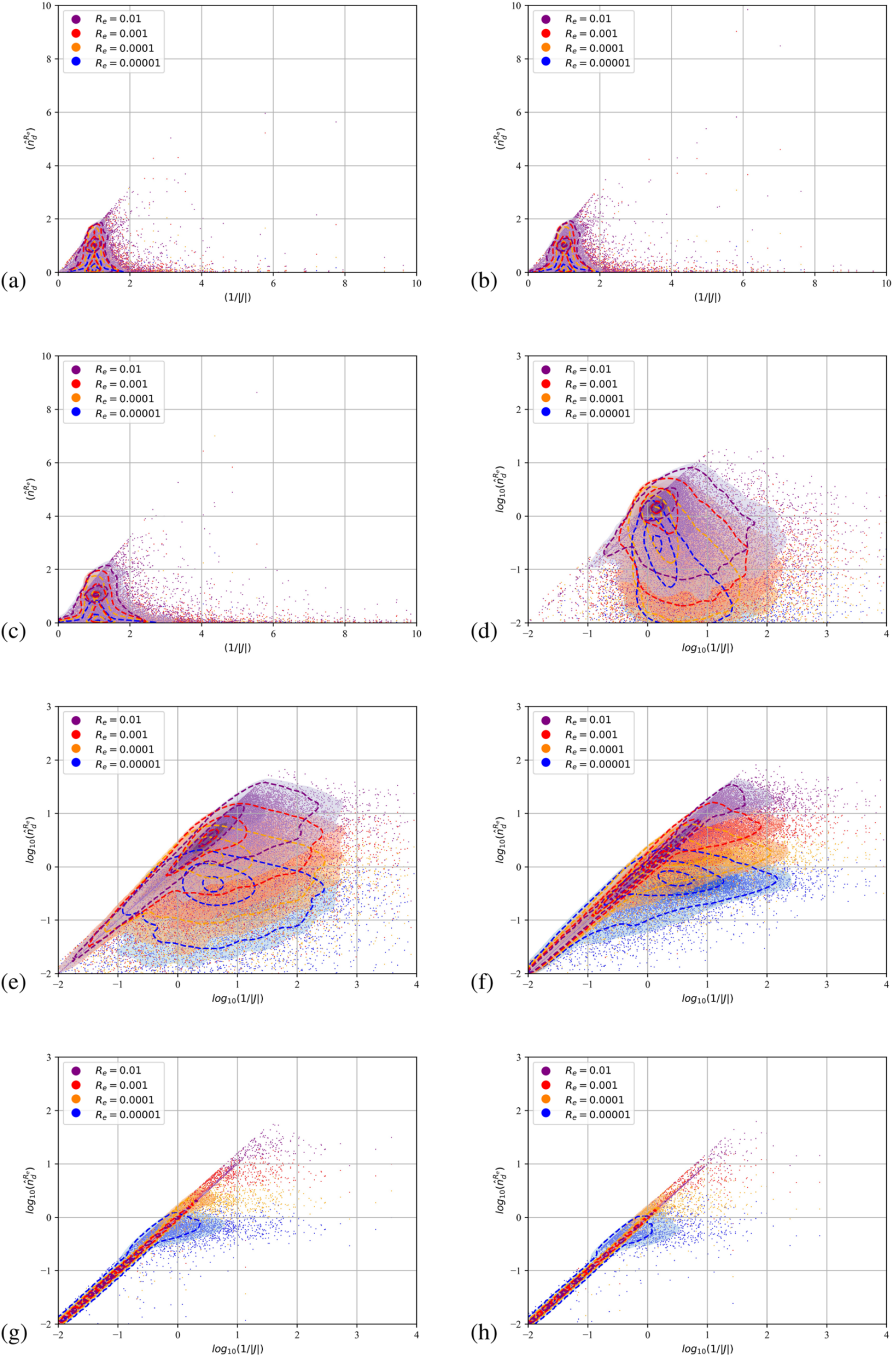
Scatter plot of the filtered FLA2 number density versus 1/*J*. Colours correspond to the filtering length. Dashed iso-lines correspond to the kernel density estimation at levels 10% 50% and 90%. Case C3DN4. $T=8$ ms. (**a**–**h**) $D_d=0.5$–$100\;\upmu$m. (**d**–**h**) are presented in logarithmic scale.

**Figure 7:**
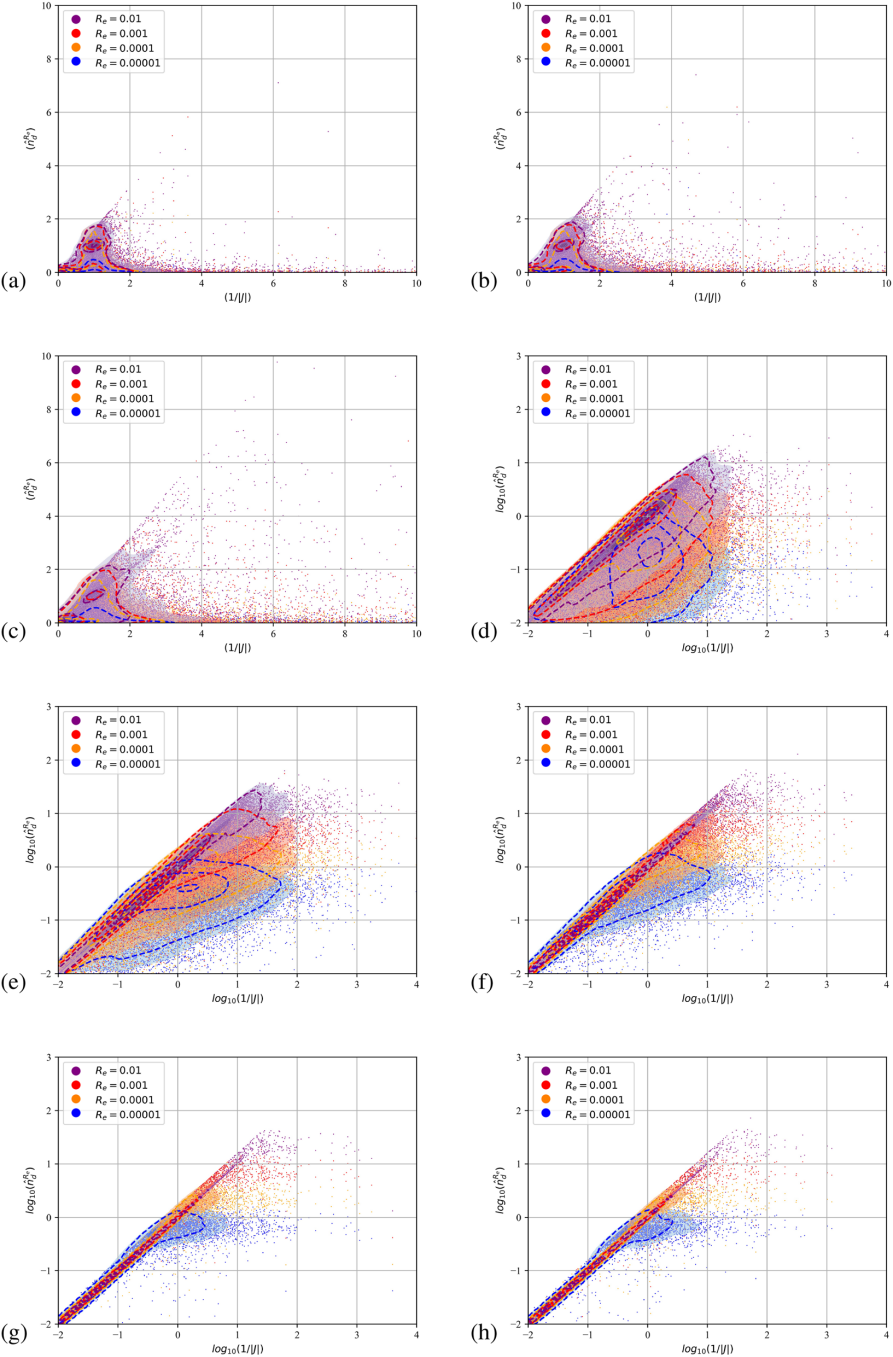
Scatter plot of the filtered FLA2 number density versus 1/*J*. Colours correspond to the filtering length. Dashed iso-lines correspond to the kernel density estimation at levels 10% 50% and 90%. Case C3DN6. $T=8$ ms. (**a**–**h**) $D_d=0.5$–$100\;\upmu$m. (**d**–**h**) are presented in logarithmic scale.

**Figure 8:**
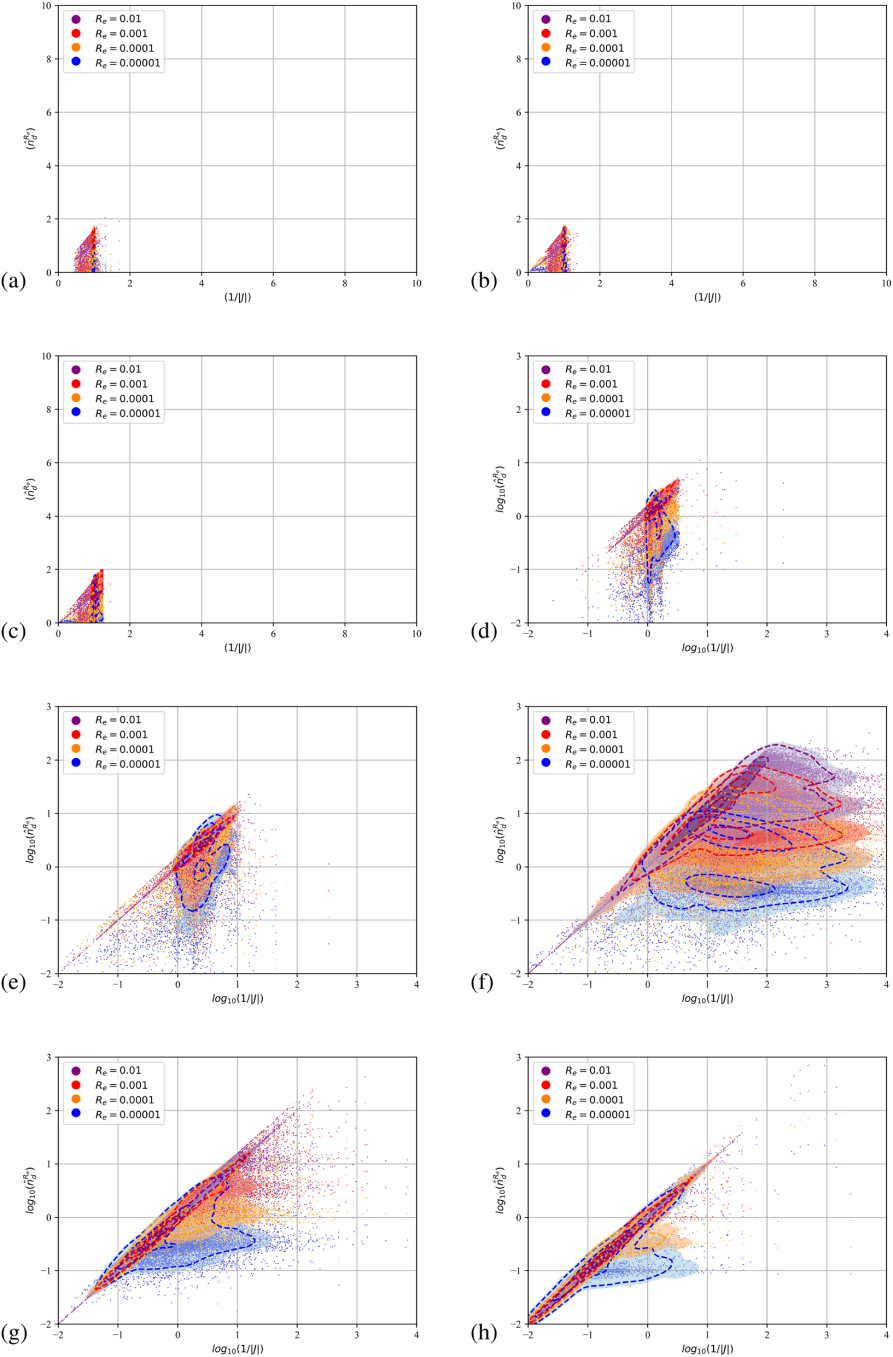
Scatter plot of the filtered FLA2 number density versus 1/*J*. Colours correspond to the filtering length. Dashed iso-lines correspond to the kernel density estimation at levels 10% 50% and 90%. Case C2DN6. $T=12$ ms. (**a**–**h**) $D_d=0.5$–$100\;\upmu$m. (**d**–**h**) are presented in logarithmic scale.

Due to their pressureless nature^45^ dispersed flows can compress to the point that particulate trajectories cross and the dispersed continua overlap. Fully Lagrangian approaches infer the particulate density by the deformation of the continuum from its initial distribution. It is expected that the transformation from the Eulerian to the Lagrangian coordinate will present a point-wise singularity at the loci of the intersections or folds. The Jacobian, being zero, cannot serve as a measure of the compression. A single surface on the Eulerian space with zero Jacobian does not not necessarily mean that particle with a finite initial separation have collapsed to a single point furnishing infinite number density. In such instances the curvature of the dispersed continuum dictates the separation between neighbouring particles. The second order FLA assumes a finite filtering length scale $R_\varepsilon$ on which the dispersed continuum is reconstructed^40^. This approach provides a number density on the caustic, i.e. the intensity of the caustic at a given length scale. The quantitative comparison of the FLA2 results with the point-wise number density provided by FLA is shown in Figs. 5, 6 and 7 for the three-dimensional DNS simulations and in Fig. 8 for the case C2DN6 at times $t=2T$. In the same figures we present the Kernel Density Estimation (KDE) of the scatter. As the Jacobian magnitude for a cloud crosses zero resulting in singular number density values, the numerically calculated $n_d$ overshoots to spurious values. For these cases FLA2 provides finite number densities that are volume averaged at an Eulerian length scale $R_\varepsilon$. These number densities correspond to the intensity of the caustic formations. As seen from Figs. 5, 6 and 7 although the 1/*J* axis provides unbounded values, the ${\hat{n}}_d^{R_\varepsilon }$ axis is bounded. Numerically, this singularity is expressed by very large spurious values whose magnitude is related to the temporal resolution of the initial value problem solver. In the Figs. 5, 6 and 7 the 1/*J* axis is cropped to a maximum value of $10^4$. As the filtering length-scale increases the limiting intensity of the caustics decreases too, as the number density is averaged on a bigger volume. The droplet nuclei with low inertia $St_t<1.0$ (see Table 3) show number densities below $10 n_d^0$ and no caustic formations are observed since the volume filtered number density ${\hat{n}}_d$ does not deviate from the point-wise number density $n_d=1/J$ (see Figs. 5, 6 and 7a–c). This behaviour presents a global character for all exhale parameters simulated, as shown in all three Figs. 5, 6 and 7. For the droplets above the critical diameter $2.94\;\upmu$m the turbulent Stokes number $St_t$ becomes larger than unity (see Table 3) and this arises as a significant deviation of the FLA2 filtered number density from the FLA result (see Figs. 5, 6 and 7d–h). The same behaviour is observed in the droplet nuclei conveyed by the laminar flow field in the low-Re case C2N2 shown in Fig. 8. For the laminar flow field case, the limiting values for the number densities appear in the Fig. 8f–h which correspond to droplets with diameter larger than the critical diameter shown in Table 3.

### Caustics intensity

From Figs. 5f, 6 and 7f it can be observed that at a length scale $R_\varepsilon =10^{-5}$ a maximum limit for the number density appears at $20 n_d^0$, at a larger length scale ($R_\varepsilon =0.0001$) this limit reduces to $10 n_d^0$ and for $R_\varepsilon =0.001$, number densities higher than $3 n_d^0$ are filtered out. For $R_\varepsilon =0.01$ we obtain the highest limiting value of $n_d^0$. This limiting value at different length scales describes the intensity of the caustics and provides a direct measure of the compression of the dispersed phase due to the large vortical structures of the flow. It also accounts for the effect of the turbulent scales on the compression and dilation of droplet nuclei. The VR seems to convey the droplet nuclei at large distances, retaining the high initial number density of the exhaled plume $n_d^0$ at large distances from the orifice. This is surprising since $n_d^0$ would have been expected to reduce due to the expansion of the cough jet.

Higher Stokes number nuclei present a ballistic behaviour^58^, which is characterised by high inertia particles moving on almost undisturbed trajectories. When crossing, these trajectories provide genuine Morse points with low *J* and low *H*. Thus, both the FLA2 and the standard FLA converge to very high number densities as shown in Figs. 5h, 6h and 7h where the scatter points and the 90% KDE probability cluster along the 1/*J* line.

In Fig. 8a–h we present the filtered number density scatter plot against the point-wise number density for the two dimensional simulations for $n=6$ exhale parameter (i.e., case C2DN6 in Table 2). As shown in Table 3, the critical droplet nuclei diameter for the formation of caustics is $16.5\;\upmu$m, which is in agreement with the appearance of a limiting maximum value for the filtered number densities shown in the Fig. 8f–h. For laminar cases, the predicted loading for the caustics approaches ${{\hat{n}}_d}=100$ for the smallest length scale shown (i.e. $R_\varepsilon =0.00001$). As expected, the point-wise number density result (x-axis) is polluted by singularities resulting to spurious point-wise number densities with $n_d>10^4$. The observed even higher intensity of the caustics for the laminar case can be attributed to the lack of small scale fluctuations that are expected to smooth the caustic fronts and damp their intensity.

## Discussion and conclusions

In this work we investigated the compression and dilation of droplet nuclei in vortex ring structures occurring in human cough configurations. These instances consist of short impulses of exhaled air and are characterised by finite exhale or injection parameters *n* dictated by the cough exhaled volume and the orifice diameter $n\sim 4V/(\pi D^3)$.

The resulting self-contained structure, can travel large distances. Numerical simulations for turbulent and laminar configurations have been carried out. The carrier phase flow field is resolved by means of second order accurate Direct Numerical Simulation (DNS) based on finite difference approach for the momentum equations and a spectral approach for the Poisson equation using FFT.

To obtain such predictions we developed a second order Fully Lagrangian Approach for Eulerian configurations in the Computational Fluid Dynamics framework. The evaluation of high order derivatives needed by the second order FLA is achieved by pre-fabricated least squares second order interpolations in three dimensions. The primary direction of the caustics was calculated by means of Singular Value Decomposition.

The effect of undulations of the carrier phase velocity due to large scale vortical and small scale turbulent structures on the concentration of exhaled nuclei and the corresponding viral load was investigated.

It was observed that the coherent vortical structure of the vortex ring as well as the turbulent fluctuations of the flow field, induce significant caustic formations in agreement to the droplet nuclei Stokes number based on the smallest time scales of the flow. Furthermore, the intensity of the caustics was observed reaching up to 20 times the initial concentration for the turbulent cases and 100 times the initial number density for the laminar configurations. This occurs at significant length scales ranging from 40 to $400\;\upmu$m, highlighting the smoothing effect of turbulent fluctuations to the intensity of the caustic fronts.

Eulerian CFD simulations^59^ and Eulerian/Lagrangian approaches^3,9^ for coughing/breathing configurations in a variety of setups suggest the reduction of the number density of the exhaled droplet clouds. In our work we observed instances of significant localised caustic formations^39^ that result in high number densities sustaining or exceeding the initial number density of the droplet nuclei clouds when exhaled.

Eulerian approaches^24,60^ can describe large-scale structures and integral parameters of dispersed flows when uniqueness of all parameters of the particulate continuum is assumed. Classes of two-particle models that allow for singularities in the phase space and intersecting trajectories^25,61–66^ have been widely applied^67,68^. Eulerian models for the particulate continuum inferred either from the kinetic equations for particles or from the equations for the probability density functions (PDF) of particles are also applicable for the modelling of inertia droplets^29,69^. Eulerian approaches, however, are bounded by the grid resolution. Dispersed phase presents fine length scales that are not correlated to the carrier phase length scales. Caustics for example occur on very thin surfaces even in fully resolved laminar flows. Standard Eulerian-Lagrangian approaches^38,70^, on the other hand, demand a prohibitive number of representative particles^71,72^. The Osiptsov method^26^, can successfully identify caustics^73^ by a small number of representative clouds. The standard FLA, however, cannot provide a prediction of the number density on the caustics. Also, the spurious values in the vicinity of the caustic singularity pollute statistical analyses^44^. For this the second order FLA was used in this work, to evaluate the intensity of droplet nuclei caustics.

The following observations conclude our findings.

1. The exhale parameter *n* was not observed to affect the concentrations of the droplet nuclei by no other means than resulting in plumes with pronounced trailing jet.
2. The trailing jet of the vortex ring conveys droplet nuclei resulting in the reduction of their number density.
3. The methodology identifies the theoretical limit (St > 1) for the formation of caustics for both laminar and turbulent simulations.
4. Both turbulent fluctuations and macroscopic VR vortical structures generate high number densities sustaining or exceeding the initial number density of the droplet nuclei clouds when exhaled.
5. Increased intensity of the caustic fronts conveyed by the large coherent vortical structures of laminar VRs was observed when compared with turbulent VRs.

## Methods

The carrier phase flow field is resolved by means of second order accurate Direct Numerical Simulation (DNS). Droplet clouds are injected with the carrier phase velocity and the Jacobian and Hessian matrices are initialised according to the initial conditions of the initial value problem of the method^40^ described in the “Supplementary Material”. Droplet clouds are distributed uniformly along the radial and the azimuthal directions of the orifice. The instantaneous dispersed phase droplet number flow rate is integrated in time. The closest integer number of droplets is then emitted, while they are subtracted from the total influx integral.

The droplets are decomposed following the domain decomposition of the carrier phase computational domain. When crossing domains, the droplet clouds are deleted from the current partition and re-injected into the new partition. In order to avoid the re-allocation of memory addresses and retain the computational efficiency of memory arrays, erased particles are flagged as empty objects which are re-used by newly injected droplet clouds.

## Supplementary Information

Supplementary Information.
