# Supplementary material for: Droplet nuclei caustic formations in exhaled vortex rings
Source: Sci Rep. 2022 Mar 10;12:3892. doi: 10.1038/s41598-022-07717-z (PMC8913841; doi:10.1038/s41598-022-07717-z)
Supplement: Supplementary file 1 — Supplementary Information. [file 41598_2022_7717_MOESM1_ESM.pdf]

Supplementary Material: Method

Droplet nuclei caustic formations in exhaled vortex rings.

Andreas Papoutsakis[1*], Ionut Danaila[2], Francky Luddens[2], Manolis Gavaises[1]

1. City University of London. School of Mathematics, Computer Science and Engineering, Department of Mechanical Engineering and Aeronautics, London, EC1V 0HB, UK.
2. Laboratoire de Mathématiques Raphaël Salem, Université de Rouen Normandie,CNRS UMR6085, 76801 Saint-Étienne-du-Rouvray, France
* Corresponding author, e-mail: andreas.papoutsakis@city.ac.uk

Author Contributions: A.P. and M.G. initiated the project. A.P. developed the model analyzed the data, performed the simulations and wrote the manuscript. F.L., I.D. and A.P. developed the parallel carrier phase solver. All authors contributed in writing and reviewing the manuscript.

Carrier phase

The momentum transport equation is discretised on a staggered Cartesian grid of $n_\theta \times n_r \times n_z$ cells with uniform size $d\theta, dr$ and dz using the second order Finite Differences (FD) implementation[1–5] in a staggered grid framework. Velocity components are evaluated on the faces of the grid, while the pressure field is evaluated at the centre of the cells. We denote by $\mathbf{Y} = \{u_\theta, u_r, u_z\}$ the carrier phase velocity field in the cylindrical coordinates[5] $\mathbf{r} = \{\theta, r, z\}$. Cartesian coordinates x_i are expressed as:

$$x_1 = rC,\ \ x_2 = rS,\ \ x_3 = z\ , \tag{1}$$

with $C = cos(\theta)$ and $S = sin(\theta)$. The governing equations are written using field variables $\mathbf{q} = \{u_\theta, r \cdot u_r, u_z\}$ as:

$$\frac{\partial q_\theta}{dt} + C_\theta = \frac{1}{Re}D_\theta - \frac{1}{r}\frac{\partial p}{\partial \theta}\ ,$$

$$\frac{\partial q_r}{dt} + C_r = \frac{1}{Re}D_r - r\frac{\partial p}{\partial r}\ ,$$

$$\frac{\partial q_z}{dt} + C_z = \frac{1}{Re}D_z - \frac{\partial p}{\partial z}\ , \tag{2}$$

where C denotes convection terms, and D diffusion terms. The Reynolds number $Re = \rho U_0 L/\mu$ is based on the non-dimensional length L taken equal to the inlet orifice diameter D and the non-dimensional velocity U_0, equal to the maximum inlet velocity. p is the pressure field and ρ is the carrier phase density (air) and μ is the dynamic viscosity of air. For the $\theta-$direction terms C_θ and D_θ are defined as:

$$D_\theta = \frac{1}{r^2}\left(\frac{\partial}{\partial r}r^3\frac{\partial}{\partial r}\frac{q_\theta}{r}\right) + \frac{1}{r^2}\frac{\partial^2 q_\theta}{\partial \theta^2} + \frac{\partial^2 q_\theta}{\partial z^2} + \frac{2}{r^3}\frac{\partial q_r}{\partial \theta},\ C_\theta = \frac{1}{r^2}\frac{\partial}{\partial r}(rq_rq_\theta) + \frac{1}{r}\frac{\partial}{\partial \theta}(q_\theta q_\theta) + \frac{\partial}{\partial z}(q_\theta q_z)\ . \tag{3}$$

For the $r-$ direction the convection and diffusion terms C_r and D_r are:

$$D_r = r\frac{\partial}{\partial r}\left(\frac{1}{r}\frac{\partial q_r}{\partial r}\right) + \frac{1}{r^2}\frac{\partial^2 q_r}{\partial \theta^2} + \frac{\partial^2 q_r}{\partial z^2} - \frac{2}{r}\frac{\partial q_\theta}{\partial \theta},\ C_\theta = \frac{\partial}{\partial r}\left(\frac{q_rq_r}{r}\right) + \frac{\partial}{\partial \theta}\left(\frac{q_rq_\theta}{r}\right) + \frac{\partial}{\partial z}(q_rq_z) - q_\theta q_\theta\ , \tag{4}$$

and for the $z-$ direction the convection C_z and diffusion D_z terms are:

$$D_z = \frac{1}{r}\frac{\partial}{\partial r}\left(r\frac{\partial q_z}{\partial r}\right) + \frac{1}{r^2}\frac{\partial^2 q_z}{\partial \theta^2} + \frac{\partial^2 q_z}{\partial z^2},\ C_\theta = \frac{1}{r}\frac{\partial}{\partial r}(rq_rq_z) + \frac{1}{r}\frac{\partial}{\partial \theta}(q_\theta q_z) + \frac{\partial}{\partial z}(q_zq_z)\ . \tag{5}$$

Pressure is evaluated on the centres of the cells and discretised by a Fourier expansion. The incompressibility assumption $\nabla.\mathbf{u} = 0$, is ensured within the fractional step method by solving a Poisson pressure correction equation[1–5].

Dispersed phase

The dispersed flow in the simulations presented here is modelled by representative parcels of droplet nuclei, driven by the Stokes drag induced by the carrier phase. If $\mathbf{U} = (u, v, w)$ is the carrier phase flow field velocity evaluated at the position of the parcel, the parcel position x_i and velocity $V_i = \dot{x}_i$ are obtained by integrating the following initial value problem:

$$\dot{x}_i = V_i \quad , \quad \ddot{x}_i = \frac{1}{St}(U_i - V_i) \ , \tag{6}$$

where St$= t_p/T_0$ is the Stokes number[6], t_p is the particle relaxation time and $T_0 = L/U_0$ the characteristic time of the flow. The second order Fully Lagrangian Approach (FLA2) is used for the integration of the mass conservation of the droplet nuclei on the Lagrangian trajectories[7]. The initial value problem for the calculation of the Hessian and the Jacobian for the second order FLA is summarised in the form of the following non-linear first order differential system:

$$\frac{\partial}{\partial t}\begin{bmatrix} J_{ij} \\ \omega_{ij} \\ H_{ijk} \\ \psi_{ijk} \end{bmatrix} = \begin{bmatrix} \omega_{ij} \\ \frac{1}{St}\left(\frac{\partial U_i}{\partial x_m} J_{mj} - \omega_{ij}\right) \\ \psi_{ijk} \\ \frac{1}{St}\left(H_{mjk}\frac{\partial U_i}{\partial x_m} + J_{mj}J_{nk}\frac{\partial^2 U_i}{\partial x_m \partial x_n} - \psi_{ijk}\right) \end{bmatrix}, \tag{7}$$

where sum is assumed over the m and n indices, with initial conditions:

$$\begin{bmatrix} J_{ij} \\ \omega_{ij} \\ H_{ijk} \\ \psi_{ijk} \end{bmatrix}_{t=0} = \begin{bmatrix} 1 \\ \frac{\partial V_i}{\partial x_j} \\ 0 \\ \frac{\partial^2 V_i}{\partial x_j \partial x_k} \end{bmatrix} \ . \tag{8}$$

The system of Equations (7) and (8) are integrated using a 3rd order Runge–Kutta method. Equations (7) and (8) together with the Lagrangian parcel kinematic Equations (6) provide the particle trajectory and the entries of the Jacobian and the Hessian matrices along a particle trajectory. To address the stiffness of the particle evolution problem for the low St number droplets, the initial value problem for the parcels is integrated by using fractional time steps within the overall carrier phase Runge–Kutta integration time step.

The evaluation of carrier phase flow field U_i, and the high-order derivatives of the carrier phase flow field $U_{i,x}$ and $U_{i,xx}$ needed in the FLA Equation (8) is obtained by a pre-fabricated least squares second order interpolation in three dimensions. This technique takes advantage of the uniform discretisation of the computational domain in cylindrical coordinates. Starting from the particle position $\mathbf{r}$ in cylindrical coordinates the closest node of the FD discretisation with coordinates $\mathbf{r}_0$ is selected and the interpolated value is calculated based on the simulated flow field solution $\mathbf{Y} = \{u_\theta, u_r, u_z\}$ on a stencil consisting of the 27 nearest neighbours. Node positions on the cubic stencil are defined by a local position vector $\mathbf{r} = \mathbf{r} + \mathbf{r}_0$, with:

$$\mathbf{r}_m = \mathbf{r}_{i_\theta, i_r, i_z} = [i_\theta d\theta, i_r dr, i_z dz], i_\theta, i_r, i_z = -1, 0, 1 \quad \text{and} \quad m = 1 \dots 27 \ . \tag{9}$$

Since the FD solution is computed on a staggered grid that consists of three discrete meshes for each one of the three momentum components, interpolation points are chosen according to the velocity component to be interpolated. A local second order polynomial interpolation of the field variables $\mathbf{Y} = Y_i$ following the θ r and z directions is assumed:

$$F_i(\mathbf{r}_m) = C_0 + C_1\theta + C_2\rho + C_3\zeta + C_4\theta^2 + C_5\theta\rho + C_6\rho^2 + C_7\rho\zeta + C_8\zeta^2 + C_9\zeta\theta, \ m = 1 \dots 27 \ . \tag{10}$$

The coefficients vector $\mathbf{C} = C_i$ with size 9 is calculated by solving the optimisation problem that minimises the magnitude of the L^2 norm of the interpolation error $R_i = Y_i(\mathbf{r}_m) - F_i(\mathbf{r}_m)$ using a Gauss-Newton approach. For this, a 27×9 Jacobian matrix $J_{m,i}$ is introduced, defined as the derivative of the polynomial value at an interpolation point m with respect to coefficient c_l, as:

$$J^i_{m,l} = \frac{\partial F_i(\mathbf{r}_m)}{\partial c_l}, \ m = 1 \dots 27 \ , \ l = 1 \dots 9 \ . \tag{11}$$

Given that the mesh is constant and uniform, $\mathbf{J}$ is constant and thus calculated analytically. The coefficients $\mathbf{C}$ are calculated by the iterative Gauss-Newton method that aims to minimise $|\mathbf{R}|$. For each iteration, the algorithm provides an improved estimate $\mathbf{C}^{n+1}$, based on an initial assumption $\mathbf{C}^n$ as:

$$\mathbf{C}^{n+1} = \mathbf{C}^n - \left[\left(\mathbf{J}^T\mathbf{J}\right)^{-1}\mathbf{J}^T\right]\mathbf{R} \ . \tag{12}$$

Matrix $\mathbf{M} = \left(\mathbf{J}^T\mathbf{J}\right)^{-1}\mathbf{J}^T$ is calculated analytically, using Mathematica[8]. The derived analytical matrix $\mathbf{M}$ is evaluated once at the start of the simulation for the specific $d\theta, dr$ and dz values and is valid for all interpolations needed throughout the simulation. The algorithm described by Equation (12) is iterated until convergence (i.e. $|\mathbf{R}| < 10^{-8}$). The converged coefficients $\mathbf{C}$ are used in the interpolation polynomial in Equation (10) for a second order estimate of field variables $\mathbf{Y}$. Derivatives $\frac{\partial Y_i}{\partial r_j}$ and $\frac{\partial Y_i}{\partial r_j \partial r_k}$ are readily available by differentiating Equation (10) analytically. To transform the interpolation result $\mathbf{Y}$, the derivatives $\frac{\partial Y_i}{\partial r_j}$ and $\frac{\partial Y_i}{\partial r_j \partial r_k}$ to Cartesian coordinates, the following implementation of the chain rule is used for the non-linear transformation in Equation (1) (i.e. $\frac{\partial^2 r_i}{\partial x_j \partial x_k} \neq 0$):

$$\frac{\partial Y_i}{\partial x_j} = \frac{\partial Y_i}{\partial r_k}\frac{\partial r_k}{\partial x_j} \, , \tag{13}$$

where sum is assumed over the k index. Second derivatives in cylindrical coordinates are transformed to Cartesian variable derivatives similarly:

$$\frac{\partial^2 Y_i}{\partial x_j \partial x_k} = \frac{\partial Y_i}{\partial r_l \partial r_m}\frac{\partial r_l}{\partial x_j}\frac{\partial r_m}{\partial x_k} + \frac{\partial Y_i}{\partial r_l}\left(\frac{\partial^2 r_l}{\partial x_j \partial x_k}\right) \, , \tag{14}$$

where sum is assumed over the l and m indices. Thus, the transformation from the cylindrical to the Cartesian derivatives is obtained by developing $\frac{\partial r_k}{\partial x_j}$ and $\frac{\partial^2 r_i}{\partial x_j \partial x_k}$:

$$\begin{bmatrix} \frac{\partial Y_i}{\partial x_j} \\ \\ \frac{\partial Y_i}{\partial x_j \partial x_k} \end{bmatrix} = \begin{bmatrix} -S/r & C & 0 & 0 & 0 & 0 & 0 & 0 & 0 & 0 & 0 & 0 \\ C/r & S & 0 & 0 & 0 & 0 & 0 & 0 & 0 & 0 & 0 & 0 \\ 0 & 0 & 1 & 0 & 0 & 0 & 0 & 0 & 0 & 0 & 0 & 0 \\ S2/r^2 & SS/r & 0 & SS/r^2 & -CS^2/r & 0 & -CS/r & CC & 0 & 0 & 0 & 0 \\ C2/r^2 & -CS/r & 0 & -CS/r^2 & CC/r & 0 & -SS/r & CS & 0 & 0 & 0 & 0 \\ 0 & 0 & 0 & 0 & 0 & -S/r & 0 & 0 & C & 0 & 0 & 0 \\ C2/r^2 & -CS/r & 0 & -CS/r^2 & CC/r & 0 & -SS/r & CS & 0 & 0 & 0 & 0 \\ -S2/r^2 & CC/r & 0 & CC/r^2 & CS/r & 0 & CS/r & S^2 & 0 & 0 & 0 & 0 \\ 0 & 0 & 0 & 0 & 0 & C/r & 0 & 0 & S & 0 & 0 & 0 \\ 0 & 0 & 0 & 0 & 0 & 0 & 0 & 0 & 0 & -S/r & C & 0 \\ 0 & 0 & 0 & 0 & 0 & 0 & 0 & 0 & 0 & C/r & S & 0 \\ 0 & 0 & 0 & 0 & 0 & 0 & 0 & 0 & 0 & 0 & 0 & 1 \end{bmatrix} \begin{bmatrix} \frac{\partial Y_i}{\partial r_j} \\ \\ \frac{\partial Y_i}{\partial r_j \partial r_k} \end{bmatrix} , \tag{15}$$

where $S2 = 2CS$ and $C2 = C^2 - S^2$. To transform the cylindrical coordinate flow field vector Y_i to the Cartesian velocity vector U_i, we use the transformation matrix $\mathbf{T}$:

$$U_i = T_{i,k}Y_k \quad with \quad \mathbf{T} = \begin{bmatrix} C & S & 0 \\ -S & C & 0 \\ 0 & 0 & 1 \end{bmatrix} . \tag{16}$$

For the linear transformation described in Equation (16), the first derivatives become:

$$\frac{\partial U_i}{\partial x_j} = T_{i,k}\frac{\partial Y_k}{\partial x_j} + \frac{\partial T_{i,k}}{\partial x_j}Y_k \, , \tag{17}$$

where sum is assumed over the k index. For the second derivatives:

$$\frac{\partial U_i}{\partial x_j \partial x_k} = T_{i,l}\frac{\partial^2 Y_l}{\partial x_j \partial x_k} + \frac{\partial T_{i,l}}{\partial x_k}\frac{\partial Y_l}{\partial x_j} + \frac{\partial T_{i,l}}{\partial x_j}\frac{\partial Y_l}{\partial x_k} + \frac{\partial^2 T_{i,l}}{\partial x_j \partial x_k}Y_l \, , \, , \tag{18}$$

where sum is assumed over the l and m indices.

In Figure 1(**a**) we present the result of the interpolation of the carrier phase $x-$axis velocity component along with the first (see Figure 1(**b**)) and second derivatives (see Figure 1(**c**)) along the $x-$axis, for a disk shaped cloud of particles conveyed by a stationary vortical flow field. The carrier phase flow field is described by the following analytical expressions:

$$u_\theta = -U_0 r \sin(2\pi k_r r)\cos(k_\theta \theta), \;\; u_r = U_0 r \cos(2\pi k_r r)\sin(k_\theta \theta), \;\; u_z = 1 \tag{19}$$

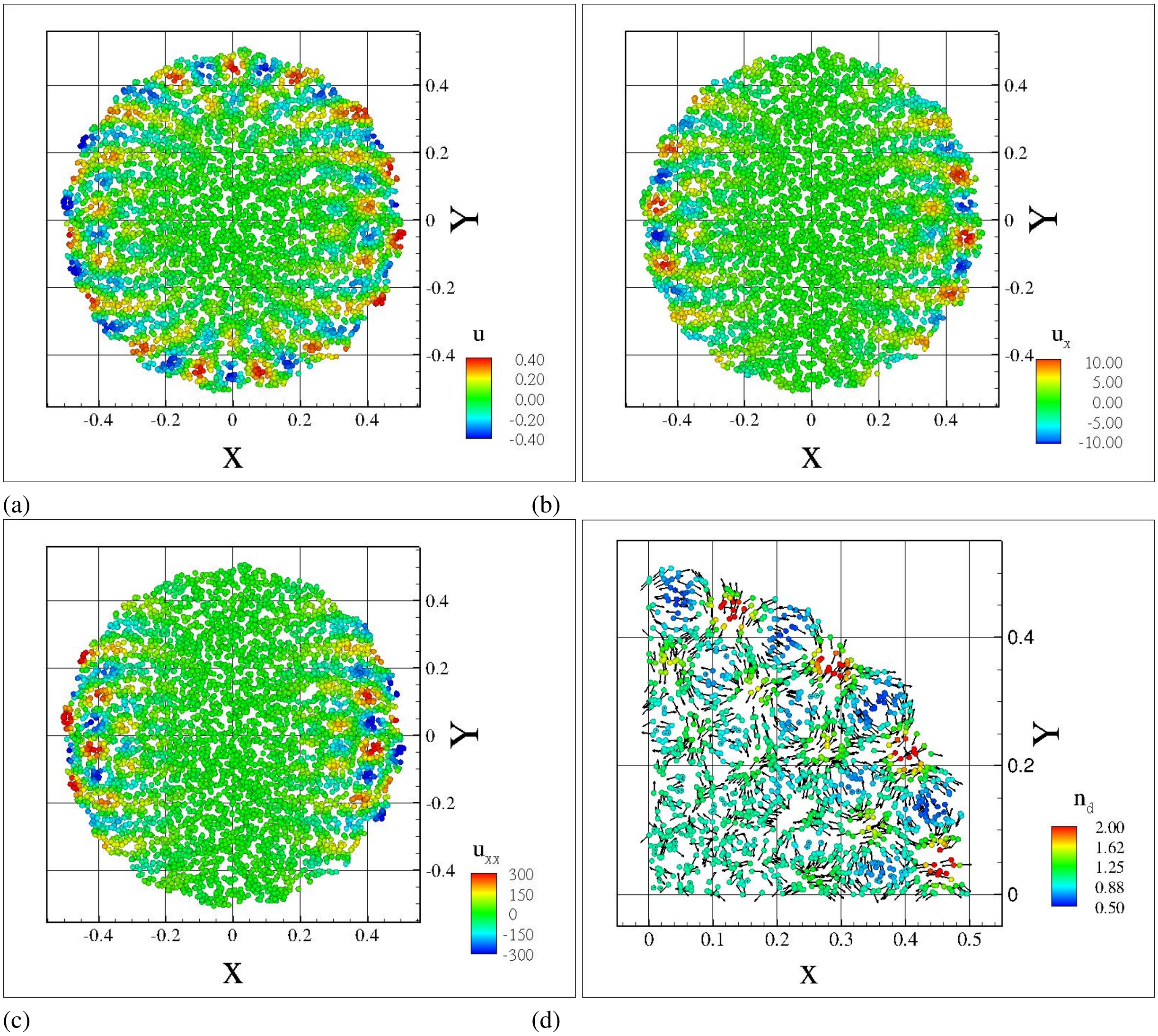

Figure 1. Distribution of a cloud of particles on the $x-y$ plane. For (a)-(c) the scatter colour corresponds to the value of the carrier phase velocity field interpolated on the particle positions. (a) Interpolated u velocity component. (b) interpolated first derivative u_x (c) interpolated second derivative u_{xx}. (d) Number density from the first order FLA. Arrows correspond to the direction vector $\vec{\varepsilon}$ of the caustic filaments (direction of the compression) as calculated by the second order FLA2.

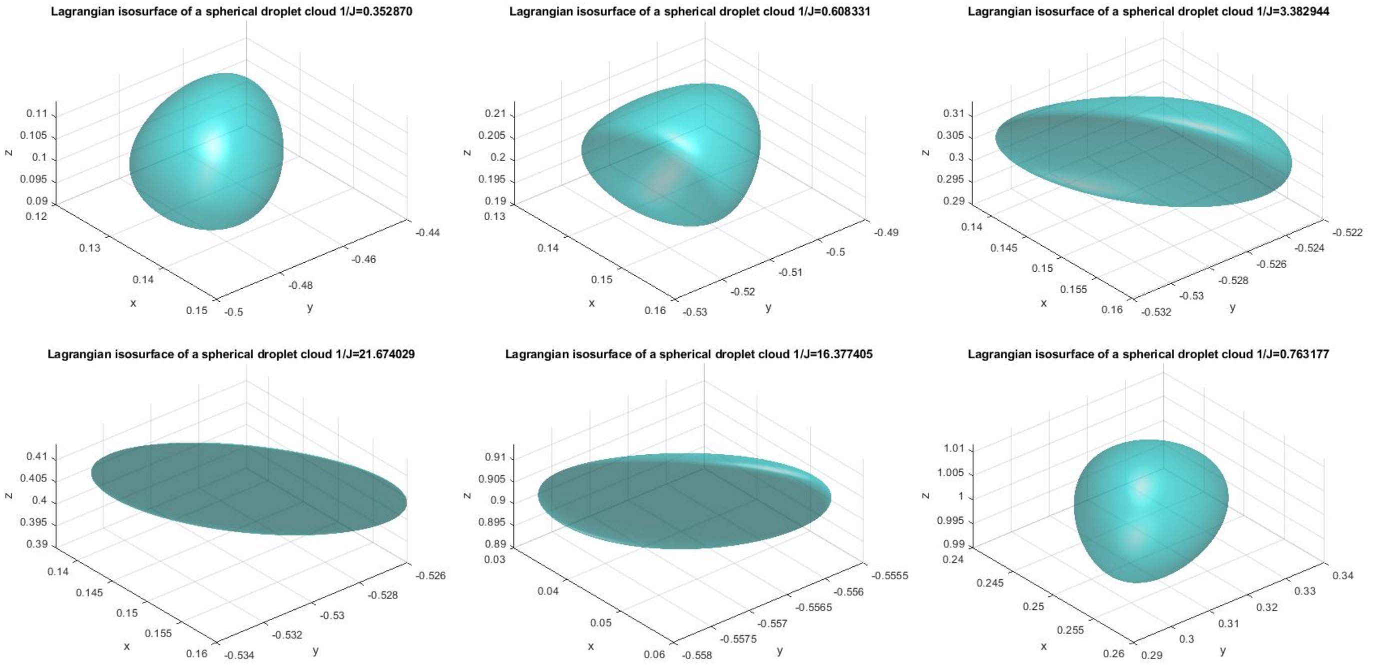

Figure 2. Temporal evolution of the deformation a droplet cloud in the Lagrangian space for six consecutive instances.

where $k_r = 5$ and $k_\theta = 16$ are the axial and azimuthal wavenumbers, respectively. The analytical field described by Equation (19) is superimposed on the inlet conditions of the DNS simulations presented in this material to emulate the inlet turbulent kinetic energy for the three dimensional DNS runs presented in the Results section. U_0, k_r and k_θ are chosen according to the inlet turbulent kinetic energy k_T and dissipation rate ε_T.

In Figure 2 we present the shape of an initially spherical cloud in the Eulerian space, as it deforms while conveyed by the flow field described by Equation (19). The deformed surface is calculated by introducing a three-dimensional Taylor expansion of the Eulerian coordinate $\mathbf{x}$ as a function of the initial Lagrangian coordinate $\mathbf{x}_0$, using the values of the Jacobian and Hessian matrices calculated by the initial value problem in Equation (7), as:

$$\mathbf{x}(\mathbf{x_0}+\delta)-\mathbf{x}(\mathbf{x}_0)=\delta\mathbf{J}+\frac{1}{2}\delta\mathbf{H}\delta \,, \tag{20}$$

with the curve vector δ laying on a sphere with radius 0.005. This representation highlights the enhanced descriptive capabilities of the second order model for the deformed continuum. The ratio between the deformed volume and the initial Lagrangian volume of the closed curve δ defines the change of the initial number density.

To derive a magnitude of the Hessian across a caustic formation, an optimisation algorithm is used to identify the primary direction of a fold. Jacobian and Hessian tensors are rotated by the three Euler angles $\vec{\varepsilon}=\{\alpha,\beta,\gamma\}$ using the following transformation:

$$\begin{bmatrix}\eta_1\\ \eta_2\\ \eta_3\end{bmatrix}=\begin{bmatrix}c_2c_3-c_1s_2s_3 & s_2c_3+c_1c_2s_3 & s_1s_3\\ -c_2s_3-c_1s_2c_3 & -s_2s_3+c_1c_2c_2 & s_1c_3\\ s_1s_2 & -s_1c_2 & c_1\end{bmatrix}\begin{bmatrix}x_1\\ x_2\\ x_3\end{bmatrix}\,, \tag{21}$$

where, $c_i=\cos(\varepsilon_i)$ and $s_i=\sin(\varepsilon_i)$. Thus, the Jacobian matrix on the rotated direction $\vec{\varepsilon}$ is given by the sum:

$$J^{\eta}_{i,j}=\frac{\partial\eta_i}{\partial\eta^0_j}=\frac{\partial\eta_i}{\partial x^0_k}\frac{\partial x_k}{\partial x^0_l}\frac{\partial x_l}{\partial\eta^0_j}\,, \tag{22}$$

where sum is assumed over the k and l indices. The Hessian in the rotated coordinate system is defined as:

$$H^{\eta}_{i,j,k}=\frac{\partial^2\eta_i}{\partial\eta^0_j\partial\eta^0_k}=\frac{\partial\eta_i}{\partial x^0_l}\frac{\partial^2 x_l}{\partial x^0_m\partial x^0_n}\frac{\partial x_m}{\partial\eta^0_j}\frac{\partial x_n}{\partial\eta^0_k}\,, \tag{23}$$

where sum is assumed over the l, m and n indices. Using Equations (22) and (23), the direction of the Caustic is identified by the Euler angle $\vec{\varepsilon}$ which maximises the normal component $H_{1,1,1}$ of the Hessian. For this a Newton-Raphson (NR) method is constructed:

$$\vec{\varepsilon}^1 = \vec{\varepsilon}^0 - \left[\frac{\partial^2 H_{1,1,1}(\vec{\varepsilon})}{\partial\vec{\varepsilon}\partial\vec{\varepsilon}}\right]^{-1}\left[\frac{\partial H_{1,1,1}(\vec{\varepsilon})}{\partial\vec{\varepsilon}}\right] . \tag{24}$$

In Equation (24), $\mathbf{X} = \frac{\partial^2 H_{1,1,1}(\vec{\varepsilon})}{\partial\vec{\varepsilon}\partial\vec{\varepsilon}}$ is the NR Hessian of the $(1,1,1)$ element of the rotated Hessian matrix H in respect to the Euler angle vector $\vec{\varepsilon}$, while $\frac{\partial H_{1,1,1}(\vec{\varepsilon})}{\partial\vec{\varepsilon}}$ is the NR Jacobian of the rotated Hessian $H_{1,1,1}$ in respect to $\vec{\varepsilon}$. Thus, the NR method in Equation (24) introduces a second pair of Jacobian and Hessian matricies akin to the NR method not to be confused with the ones introduced in the second order FLA method.

For an non-deformed dispersed continuum, there is no preferential direction. Thus, $\mathbf{X}$ can be singular and the preferential direction can have one or two degrees of freedom and $\mathbf{X}$ cannot be inverted. For this reason, a pseudo-inverse is used instead, which ensures that the optimisation problem accepts a set of solutions. The pseudo-inverse of the Hessian $\mathbf{X}^+$ of $H_{1,1,1}(\vec{\varepsilon})$ with respect to the Euler angle $\vec{\varepsilon}$ is calculated using the Singular Value Decomposition (SVD) algorithm[9] which provides the diagonalisation arrays U and V for X as $\mathbf{X} = \mathbf{U}\Sigma\mathbf{V}^T$. The pseudo-inverse is then calculated from U, V and the diagonal matrix Σ as:

$$\mathbf{X}^+ = \mathbf{V}\Sigma^+\mathbf{U}^T . \tag{25}$$

Σ is inverted explicitly as $\Sigma^+_{i,i} = 1/\Sigma_{i,i}$. The non-diagonal entries of Σ^+, and also the diagonal entries for which $\Sigma_{i,i}$ is zero, are replaced with zero.

According to the Osiptsov method (FLA),[10] the absolute value of the determinant of the Jacobian $J = |det(\mathbf{J})|$ provides a measure for the parcel number density of n_d according to:

$$n_d = n_d^0/J . \tag{26}$$

The result of Equation (26) for the cloud dispersed by the flow field Equation (19) is shown in the Figure 1 (see Figure 1(**c**)). In the same figure, the orientation vector $\vec{\varepsilon}$ obtained by the algorithm Equation (21) is shown. The orientation vector shows the direction of the dilation and compression regions for this specific case.

The second order FLA provides a spatially defined number density for a volume with characteristic length $\Delta = 2R_\varepsilon$ on the Eulerian field (to be compared with Equation (26)):

$$\hat{n}_d = \begin{cases} n_d^0 \frac{2}{\sqrt{J^2+2HR_\varepsilon}+\sqrt{J^2-2HR_\varepsilon}} & \text{if } J^2 - 2HR_\varepsilon > 0 \\ n_d^0 \frac{\sqrt{J^2+2HR_\varepsilon}+\sqrt{-J^2+2HR_\varepsilon}}{2R_\varepsilon H} & \text{if } J^2 - 2HR_\varepsilon < 0 \end{cases} . \tag{27}$$

For $J >> \sqrt{2HR_\varepsilon}$, the dispersed phase is diluted and the above expression simplifies to the classical FLA expression in Equation (26).

The spatially averaged number density can also provide a measure of the surface droplet loading as $R_\varepsilon \hat{n}_d$, if a two-dimensional structure of the compression filament[7] is assumed. By introducing a minimal length scale R_ε^{min}, we guarantee that at least one particle lays within the filtering volume. Although the method is expressed in terms of the number-density relative to the initial concentration of the droplet cloud, R_ε^{min} is related to the actual particle loading of the flow. For an initial particle load n_d^0, the initial length-scale for the un-deformed dispersed continuum is $l_0 = (n_d^0)^{-1/3}$. For the deformed continuum, assuming again a primary compression direction, the minimal length scale becomes $R_\varepsilon^{min} = 1/(\hat{n}_d n_d l_0^2)$.

References

1. Danaila, I. & Helie, J. Numerical simulation of the postformation evolution of a laminar vortex ring. *Phys. Fluids* **20**, 073602 (2008).
2. Orlandi, P. *Fluid Flow Phenomena: A Numerical Toolkit* (Kluwer Academic Publishers, Dordrecht, 1999).
3. Verzicco, R. & Orlandi, P. A finite–difference scheme for three–dimensional incompressible flow in cylindrical coordinates. *J. Comput. Phys.* **123**, 402–414 (1996).

4. Danaila, I., Dušek, J. & Anselmet, F. Coherent structures in a round, spatially evolving, unforced, homogeneous jet at low Reynolds numbers. *Phys. Fluids* **9**, 3323–3342, DOI: 10.1063/1.869446 (1997).

5. Danaila, I., Luddens, F., Kaplanski, F., Papoutsakis, A. & Sazhin, S. S. Formation number of confined vortex rings. *Phys. Rev. Fluids* **3**, 094701, DOI: 10.1103/PhysRevFluids.3.094701 (2018).

6. Sazhin, S. *Droplets and Sprays* (Springer, London, 2014).

7. Papoutsakis, A. & Gavaises, M. A model for the investigation of the second-order structure of caustic formations in dispersed flows. *J. Fluid Mech.* **892**, A4, DOI: 10.1017/jfm.2020.176 (2020).

8. Wolfram Research, Inc. Mathematica 8.0.

9. Press, W. H., Teukolsky, S. A., Vetterling, W. T. & Flannery, B. P. *Numerical Recipes 3rd Edition: The Art of Scientific Computing* (Cambridge University Press, USA, 2007), 3 edn.

10. Osiptsov, A. Lagrangian modelling of dust admixture in gas flows. *Astrophys. Space Sci.* **274**, 377–386 (2000).
